# How many roads must a Malaysian walk down? Mapping the accessibility of radiotherapy facilities in Malaysia

**DOI:** 10.1371/journal.pone.0213583

**Published:** 2019-03-21

**Authors:** Noorazrul Yahya, Nur Khalis Sukiman, Nani Adilah Suhaimi, Nor Aniza Azmi, Hanani A. Manan

**Affiliations:** 1 Diagnostic Imaging & Radiotherapy Programme, Faculty of Health Sciences, National University of Malaysia, Kuala Lumpur, Malaysia; 2 Department of Radiology, Faculty of Medicine, National University of Malaysia, Kuala Lumpur, Malaysia; Federal University of Rio de Janeiro, BRAZIL

## Abstract

**Background:**

The accessibility to radiotherapy facilities may affect the willingness to undergo treatment. We sought to quantify the distance and travel time of Malaysian population to the closest radiotherapy centre and to estimate the megavoltage unit (MV)/million population based on the regions.

**Materials & methods:**

Data for subdistricts in Malaysia and radiotherapy services were extracted from Department of Statistics Malaysia and Directory of Radiotherapy Centres (DIRAC). Data from DIRAC were validated by direct communication with centres. Locations of radiotherapy centres, distance and travel time to the nearest radiotherapy were estimated using web mapping service, Google Map.

**Results:**

The average distance and travel time from Malaysian population to the closest radiotherapy centre were 82.5km and 83.4mins, respectively. The average distance and travel were not homogenous; East Malaysia (228.1km, 236.1mins), Central (14.4km, 20.1mins), East Coast (124.2km, 108.8mins), Northern (42.9km, 42.8mins) and Southern (36.0km, 39.8mins). The MV/million population for the country is 2.47, East Malaysia (1.76), Central (4.19), East Coast (0.54), Northern (2.40), Southern (2.36). About 25% of the population needs to travel >100 km to get to the closest radiotherapy facility.

**Conclusion:**

On average, Malaysians need to travel far and long to reach radiotherapy facilities. The accessibility to radiotherapy facilities is not equitable. The disparity may be reduced by adding centres in East Malaysia and the East Coast.

## Introduction

Cancer patients are often conflicted with psychological, social and economic distress associated with their diagnosis [1, 2]. Such distress is further compounded by the burden to travel to access medical facilities. This is especially pressing for radiotherapy as the nature of fractionated radiotherapy requires patients to travel to the centres more frequently. For many patients, travel to cancer treatment is described as an inconvenience and a practical hardship [3, 4]. It is not surprising that studies have found that distance plays a major role in acceptance of radiotherapy, whereby its utilisation relies fairly on convenience and proximity to the radiotherapy facilities [5–9]. Reducing geographical distance may be a way to achieve a more equitable access to radiotherapy among patients.

It was estimated that more than half of cancer diagnoses worldwide occur in low- and middle-income countries and its incidence is expected to rise significantly within the next 20 years [10]. Similar pattern was seen in Malaysia [11, 12]. About 50% of cancer patients may require radiotherapy as part of their treatment. However, there is currently a shortage in radiotherapy services within these low and middle-income countries, including Malaysia [11]. Studies have highlighted the potential gains in terms of survival and local control achieved for patients in these countries are attributed to optimal supply of radiotherapy [13, 14].

In this study, we quantified the accessibility of radiotherapy facilities in Malaysia via two measures. First, we quantified the distance and travel time of Malaysian population to the closest radiotherapy centre. Second, we calculated the megavoltage unit (MV)/million population by region to provide a better estimate of accessibility compared to European standard megavoltage unit (MV)/million.

## Materials and methods

### Radiotherapy facilities

The radiotherapy facilities offered by every radiotherapy centre were obtained from the IAEA’s Directory of Radiotherapy Centres (*dirac*.*iaea*.*org)*. Then, the details were updated by contacting the personnel (usually medical physicists or radiation therapists) from all radiotherapy centres in Malaysia via email, calls and instant messaging. Data were obtained to update the changes in facilities available in radiotherapy centres including additional number of MV units. To ensure usability of this analysis in the near future, we also add MV units which are now being installed. The locations of the centres were coded using web mapping service, Google Maps (Menlo Park, California)

### Malaysian population

We contacted Department of Statistics Malaysia (DOSM) to obtain demographic information based on subdistricts. The DOSM has since allowed the data to be accessed on their website (*dosm.gov.my*).

**Fig 1:**
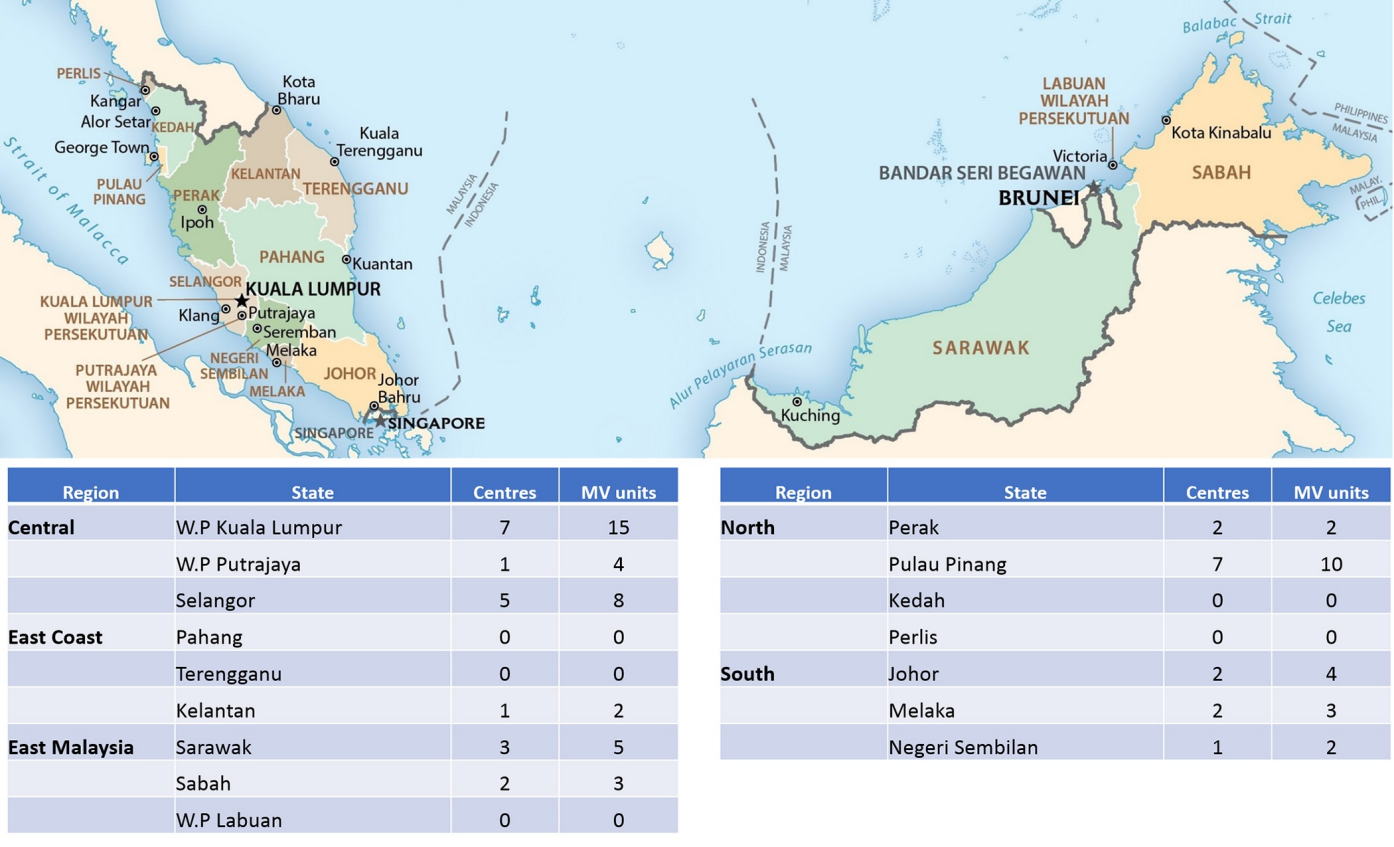
Regions in Malaysia and location of radiotherapy centres. The map was derived from a map available in Public Domain (CIA Maps, www.cia.gov).

Regions in Malaysia are defined as East Malaysia (including states and Federal Territory in Borneo, i.e. Sabah, Sarawak and federal territory of Labuan), Northern Region (Perlis, Kedah, Penang, Perak), East Coast Region (Kelantan, Terengganu, Pahang), Central Region (Selangor, and federal territories of Kuala Lumpur and Putrajaya) and Southern Region (Melaka, Johor and Negeri Sembilan) (Fig 1).

### Distance and travel time

The route planner under "Get Directions" in Google Maps, was used to determine the distance and travel time from each subdistrict in Malaysia to the closest radiotherapy centre. The points located at the geographical centre of the subdistricts were selected as the starting point and the centres’ entrance as the end points. To standardise the travel time collection and to prevent differences in traffic condition, the planner was set to estimate travel time when the travel start at 9.00 am on Wednesday during normal working days (i.e. not a public or school holiday) using a car. In instances where the subdistricts have no land route access to the radiotherapy centre (i.e. patients may need to travel via boats or small charter flights), the distances and travel times were estimated to be similar to the highest distances and travel times in the subdistrict. This is a reasonable assumption because districts with no road access to the city are mostly remote.

The average distance and travel time per population was calculated using this formula;

$$Average\;distance/travel\;time=\frac{\sum (population\;in\;each\;subdistrict\times distance\;or\;travel\;time)}{Total\;Malaysian\;population}$$

The same calculation was used to estimate the average distance and travel time for each region of the country.

Another option of calculating the distance is by calculating the distance radius from each subdistrict to the closest radiotherapy facility. However, we decided to use actual travel distance and actual time based on Google Maps real-time estimates as these measures give a more accurate estimation of time and distance taking into account the impact of congestion, road quality and topography.

### Megavoltage unit per million population

The megavoltage unit per million population (MV/mil) was calculated for the country and the regions. We do not include other types of radiotherapy (intraoperative, brachytherapy) because of limited clinical utility of these machines.

### Ethical statement

This study does not contain any experiment involving human or animal subjects performed by any of the authors.

## Results

**Fig 2:**
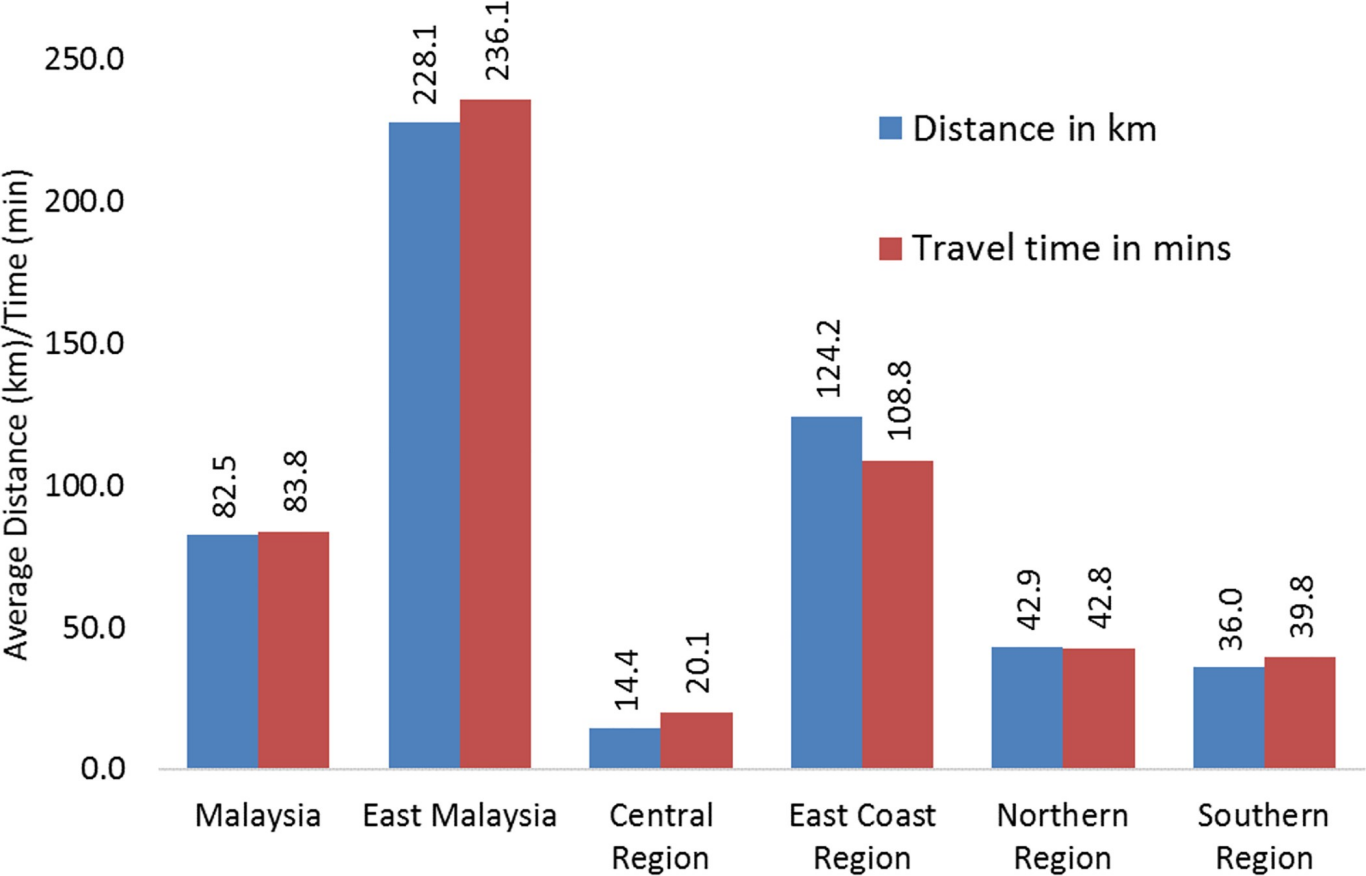
Average distance and travel time to the closest radiotherapy facility and to the closest government/ public university facility in Malaysia and in its regions.

58 megavoltage machines are available in Malaysia in 30 centres (Fig 2) as of 20/2/2019. Even though the total number of megavoltage in Malaysia is almost similar to the one reported in DIRAC database (55 machines), there are differences in the capacity in individual hospital with 7 centres underreported and 5 overreported their capacity. Less than half (23/58) of these machines are in government or public university facilities.

**Fig 3:**
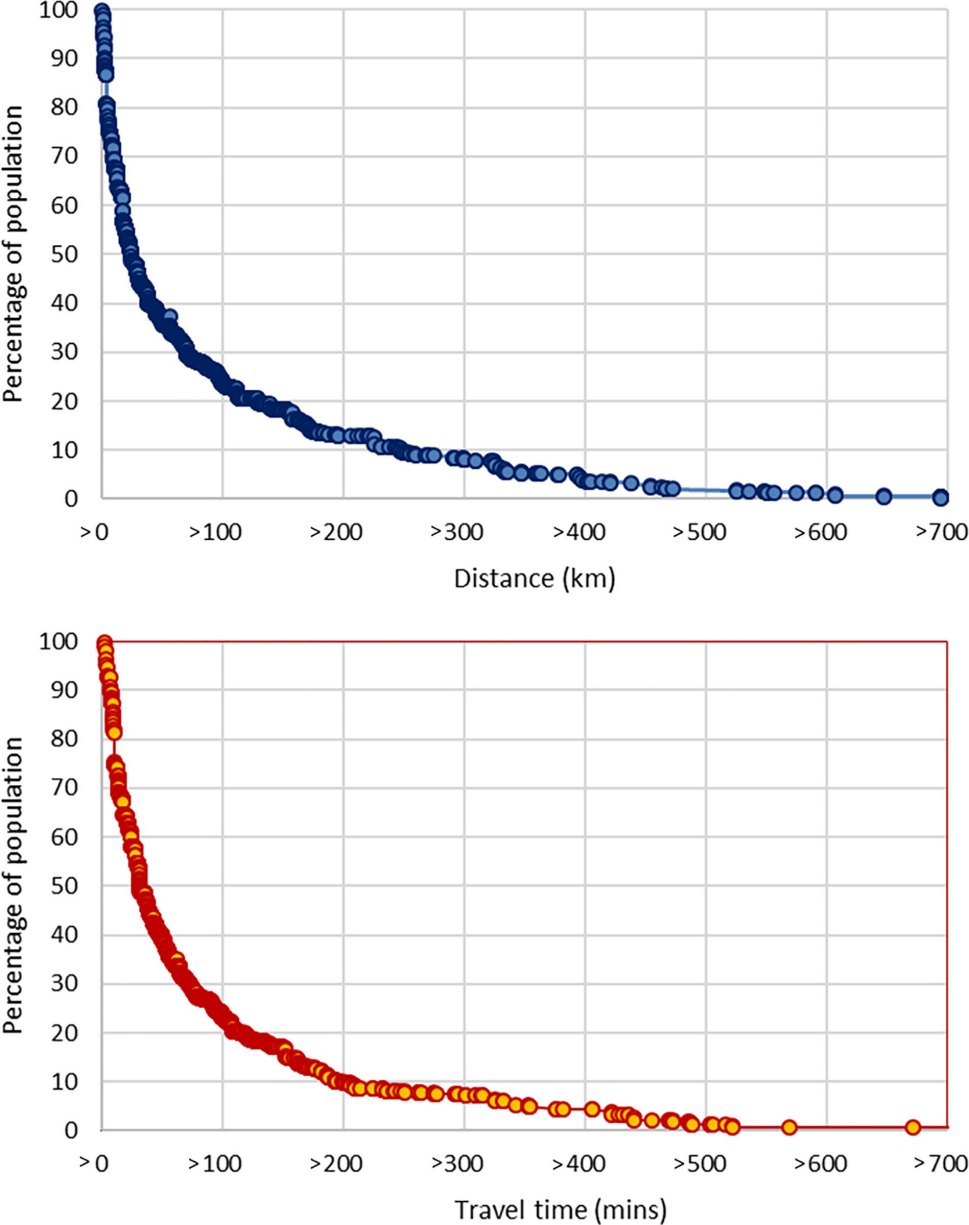
Percentage of population and a) the travel distance (km) and b) travel time (mins).

**Fig 4:**
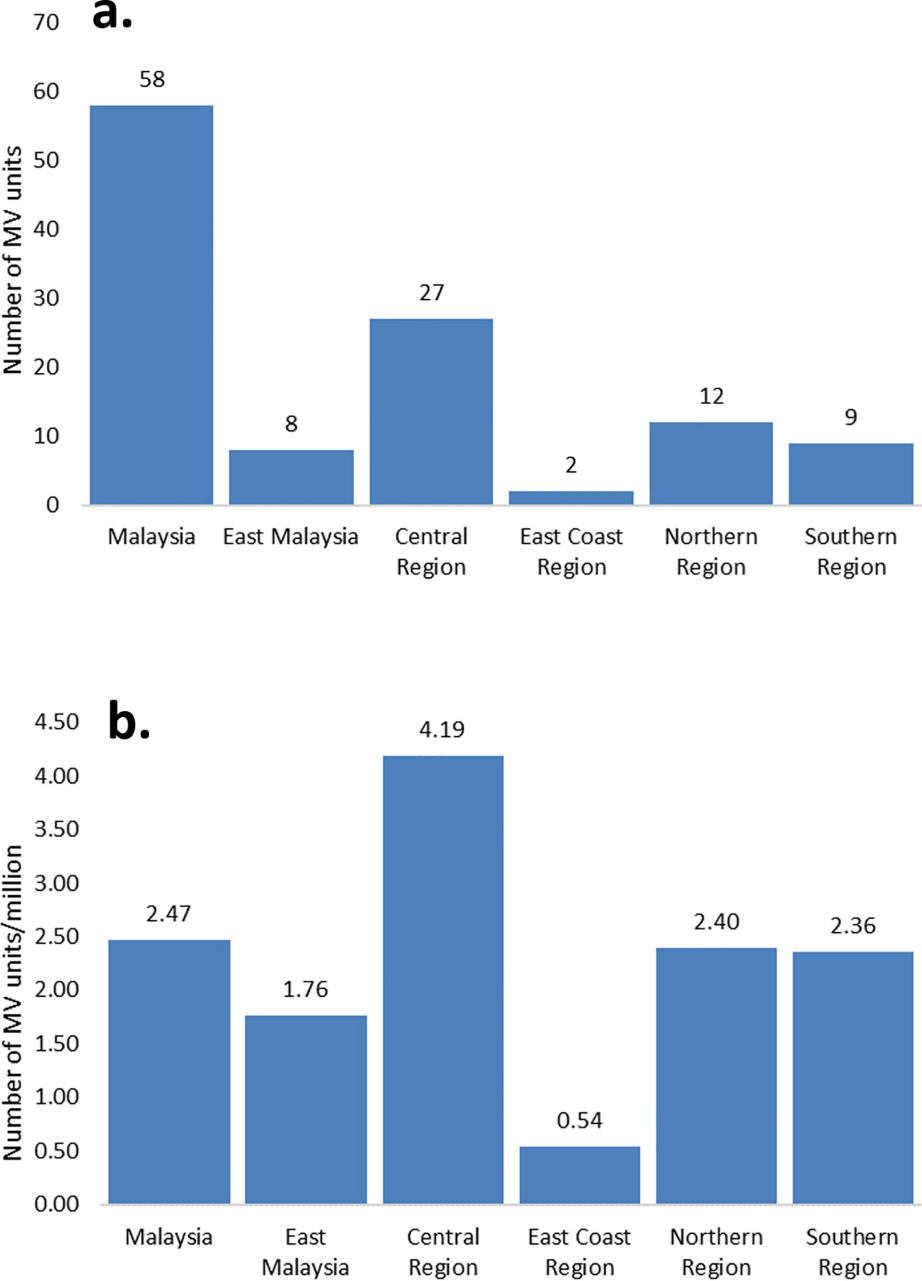
a) Number of MV units in Malaysia and its regions. b) MV units/million of population in Malaysia and in its regions.

The average distance and travel time for Malaysian population to their closest radiotherapy centre was 82.5 km and 83.4 mins, respectively (Fig 3). The average distance and travel time are not homogenous; East Malaysia has the highest average (228.1 km, 236.1 mins) while Central Region has the lowest (16.7 km, 22.6 mins). The East Coast Region averages more than 100 km and 100 mins (124.2 km, 108.8 mins) whilst the Northern Region (42.9 km, 42.8 mins) and Southern Region (36.2 km, 39.0 mins) both average significantly less than 100 km and 100 minutes. The closest government/public university facilities are more also shown for comparison (Fig 3). About 25% of the populations need to travel >100 km and about 100 minutes to reach the closest radiotherapy centres (Fig 4). The number is highest for East Malaysia populations.

The MV/million population for the country is 2.47 (Fig 4B), East Malaysia (1.76), Central (4.03), East Coast (0.54), Northern (2.40), Southern (2.36).

## Discussion

The present study is the first study to report accessibility of radiotherapy facilities in Malaysia based on the distance and travel time. Despite previous reports made on quantification of accessibility in different geographical distributions and populations in North Queensland [15] and British Columbia [16], no previous data was available on accessibility of radiotherapy in Malaysia. We found that, on average, Malaysia population needs to travel a large distance to access radiotherapy facility. The accessibility is also heterogenous across regions.

About a quarter of Malaysians need to travel more than 100 km to the closest radiotherapy centre. This may reduce the likelihood of many patients diagnosed with cancer to receive radiotherapy treatment due to the distance. Lin et al. observed that cancer patients traveling more than 50 miles (80.5 km) were less likely to receive cancer treatment compared with those traveling shorter distance [17]. This adds complexities to patient’s decision making processes which are already conflicted by factors such as fear and anxiety about radiotherapy, perceived side effects, post-traumatic stress disorder and high prevalence of complementary and alternative medicine in Asian population [18–20].

These patients who are living far from radiotherapy are also likely come from smaller towns and villages with significantly lower income. Populations in the East Malaysia, for example, have about half of the median household income compared to those residing in Kuala Lumpur [21]. This adds further financial strain especially as they need to travel further to access the government/public university facilities which provide services at significantly lower costs. A good and comprehensive cancer treatment is equally the right of the poor and underprivileged, therefore it should be our aim to ensure equitability of public cancer care services [22, 23].

The geography of the East Malaysia poses a unique challenge for the accessibility to radiotherapy centres. The states within Borneo are mountainous and sparsely populated, thus, many centres are located only in the two capital cities, Kuching and Kota Kinabalu. Despite the high technological requirements associated with radiotherapy, Arenas et. al have shown the feasibility of radiotherapy decentralisation [24]. The clear advantage of a closely located radiotherapy facility for the comfort of the patients and the economic benefit with reduced transport costs may suggest the advantage of a satellite radiotherapy unit in parts of East Malaysia [25]. There are also some advantages of having centralised care for radiotherapy (i.e. centres are located in the cities and provide access to all patients willing to travel) including lower cost of installing radiotherapy machine [26]. However, a lack of appropriate treatment closer to home may reduce the utilization of radiotherapy and this may increase the risk of recurrence [27]. If the centralised centre approach is to be adopted due to financial constraints, better accommodation options should be planned for out-of-town patients to offset the costs of accessing care for both patients and their carers especially for paediatric patients [28, 29].

In the East Coast region where the number of MV units per million is low, the installation of a new centre may be appropriate to reduce the distance and travel time for the region. To determine the optimal location, Santibanez et al. have proposed a data-driven mean to quantitatively evaluate alternative location configurations for optimum benefit in term of access [16].

The number of megavoltage units in relation to the population is still very low, significantly lower than European countries (median 5.3 MV/mil) [30]. Rosenblatt et al. in an IAEA study observed that radiotherapy utilisation rate in Malaysia did not differ significantly from that found in higher income countries [31]. The study, however, focused on a regional data with only 10% coverage which may not be representative of the actual figure. This observation matches our estimate of MV units per million of population in the Central region which is at 4.19, better than IAEA’s recommendation of 4 MV units per million population. Regions other than the Central Region have lower than the overall MV/mil in Malaysia. With the increasing incidence of cancer [10], the demands of radiotherapy will be increasing alarmingly and increase deficit in radiotherapy services if their capacities are not increased.

There are some limitations of this study which may require clarification. First, we focus on external beam megavoltage radiotherapy machines only omitting Gamma Knife which utilises Cobalt-60, brachytherapy and intraoperative radiotherapy machines. The reasons for the exclusion are two-fold. First, these machines are more specialised which utilisations were limited to certain indications. Second, to date, centres offering Gamma Knife are limited to 3 centres (all in Central region) and intraoperative radiotherapy are limited to 7 centres (5 in Central, 1 North and 1 East Coast). Second, we also note that the DIRAC database provides inaccurate number of MV units which may relate to the self-reported and voluntary nature of the database. In this study, we ensure the correct number of MV machines by directly contacting the centres.

In conclusion, Malaysians needs to travel far and long distance to reach radiotherapy facilities. The accessibility to radiotherapy facilities is not equitable. We suggest that this disparity may be reduced by adding centres in East Malaysia and the East Coast.
